# Books Review

**Published:** 1869-09

**Authors:** 


### Books Review.

*Niemeyer's Text-Book of Practical Medicine, in two volumes. Translated from the German by George H. Humphries, M. D., and Charles E. Hackley, M. D.* D. Appleton & Company, New York.

It would at first thought seem quite unnecessary, at the present time, to furnish the American profession with a new work upon the Theory and Practice of Medicine. We commenced examining the present volumes with no expectation of being able to find anything of special interest not already furnished in our previous works upon the subject. We have not discovered anything especially new, though the work is certainly up with all the advances of the last few years, and we most fully concur in the translators statements, that "Professor Niemeyer's volumes present a concise and well digested epitome of the results of ten years of carefully recorded clinical observations by the most illustrious medical authorities of Europe, together with many valuable and practical deductions regarding the causes of disease and the application of remedies, such as we believe have not as yet been assembled in any single work."

The attention given to the nature, causes and natural termination of disease, is a most attractive feature of this work. We think no one can fail of being attracted by the manner in which the pathology of disease is presented. We have examined the work with the greatest satisfaction, and find in it much more to commend than we had supposed possible. In presenting recent views of disease, the fallacies of former doctrines is shown with such clearness and force of reasoning, as well as proved by such array of facts as to make conviction certain. We certainly believe that this work will receive the approval of all, and will become an especial favorite both with students and practitioners of medicine. It is extensive in its range, complete in its description of disease and of the means of cure, and above all, it is unusually satisfactory in its descriptions of the causes, conditions, and terminations of disease. The publishers have also done their part in a most tasty and substantial manner, and we bespeak for this work, upon practical medicine, the highest position both as a text book for students and a guide to practitioners.

---

*Fowne's Chemistry. Tenth Edition.* By ROBERT BRIDGES, M. D., Phila.

In the present edition, this work presents two hundred and fifty-seven pages additional to those of the previous one, so that it now numbers in all eight hundred and fifty-seven pages; twenty-five new plates have been inserted, while an equal number of old ones are withdrawn. Ten years have elapsed since the publication of the former edition, and the progress made in the science during this period, has rendered an extended revision of its teaching and terminology, imperative. Greater advance has been made in this science, during the past few years, than ever before in the same length of time. The abundance and variety of its facts, increase yearly with the increasing number of experimental inquirers, while the acute penetration which is exercised in chemical research is plainly indicated by its recent disclosures. In the last edition of this work nothing is said of the spectroscope, now three pages and two plates are given to the subject; and in a future edition we shall probably see this occupying a distinct department of the work. The French decimal system of weight and measure is used in this volume, and the Centigrade Thermometer has the precedence, while the degrees of Fahrenheit are still retained enclosed in parentheses. We notice in the new nomenclature used, two departures are made from the general rule in giving lead, nitrate, etc., in place of plumbic nitrate, copper sulphate for cupric sulphate, which may be a favor to the English language but hardly conduces to the unity of scientific terminology; manganoso-manganic oxide is also given instead of mangano-manganic oxide. The chapter on the general principles of chemical philosophy is quite good; and we recommend that section on atomic weights to any who are wishing for an intelligent explanation of the changes introduced by the new system. The system of notation throughout the book has been made correspondent with present acceptation, and the old has not been retained to confound the student. Very many recently obtained facts concerning the rare elements are given, and much has been added in the giving of the chemical reactions. Crystallography, we expect, will be made more determinative and definite when our students shall require it. The method of estimating electro-motive power and quantity, will be new to most of its readers, while the department of Organic Chemistry will hardly be recognized, so extended has been its revision. Part fourth, on Animal Chemistry, has been enlarged and almost entirely re-written, and is of great value to the medical student. Notwithstanding the labor expended in the enlargement of the volume, its price remains as before.

The Colleges of Pharmacy lately instituted in this country require of their students particular attention to a course of chemical study. Manufacturers, who have entrusted their chemical operations hitherto to uneducated artisans, begin to see the economy of employing a skilled chemist to direct such affairs, while a knowledge of the science is diffusing among the public who recognize its utility in every-day experience. We are happy to see that chemical science, in its recent eminent advancement, has not left in the rear this work, which was so long, and is now, the best of our text books in general chemistry.

---

*Report on Excisions of the Head of the Femur.* Circular No. 2; Surgeon General's Office.

Great honor is reflected upon the Surgery of the United States by the publication of such model reports as repeatedly come from our Surgeon General's Office. In this last report, which is by Geo. A. Otis, an excellent and interesting historical review of the operation of excision of the head of the femur precedes the main report, in which latter are presented sixty-three authenticated cases which occurred in the war of the rebellion, of which forty-eight were performed by Surgeons of the Union Army, and fifteen by those of the Confederate. The operations are included in three divisions: primary, intermediate and secondary; primary operations include those performed within twenty-four hours after the injury; intermediate, those performed during the existence of inflammatory action in the parts; secondary, these performed after inflammation had subsided. There are thirty-two primary excisions, twenty-two intermediate, nine secondary. Two primary operations were successful, two intermediate and one secondary, while the average duration of life in the unsuccessful cases were, of the primary, seven days; of the intermediate, twelve and one-half days; of the secondary, sixteen days. We notice that the whole number of hip-joint excisions which have been made in military surgery, is given as eighty-five, of which twelve only were before our late war and ten since. The second half of the work thoroughly canvasses the eligibility of the operation of excision—first, as regards temporization; second, as compared with amputation, in which the collection of facts is most complete. In the concluding observations by the writer he says that, although the numerical ratios give one-half per cent. in favor of amputation, still such close reckoning, with such varied condition of the patient and sources of testimony, ought not to be considered as decisive in its favor.

"Primary excision of the head or upper extremity of the femur should be performed in all uncomplicated cases of gunshot fracture of the head or neck. Intermediate excisions are indicated in similar cases where the diagnosis is not made out till late, and also in cases of gunshot fracture of the trochanters with consecutive arthritis. Secondary excisions are demanded by caries of the head of the femur or secondary involvement of the joint, resulting from fractures in the trochanteric region or wounds of the soft parts in the immediate vicinity of the joint. Expectant treatment is to be condemned in all cases in which the diagnosis of direct injury to the articulation can be clearly established."

"Amputation at the hip-joint for gunshot injury, notwithstanding its great fatality, cannot be altogether discarded, and should be performed under the following circumstances: 1. When the thigh is torn off, or the upper extremity of the femur comminuted with great laceration of the soft parts, in such proximity to the trunk that amputation in the continuity is impracticable. 2. When a fracture of the head, neck, or trochanters of the femur is complicated with a wound of the femoral vessels. 3. When a gunshot fracture, involving the hip-joint, is complicated by a severe compound fracture of the limb lower down, or by a wound of the knee-joint. There are two other possible contingencies under which primary or early intermediate coxo-femoral amputations for injury, may be admissible: 1. When, without fracture, a ball divides the femoral artery and vein near the crural arch; 2. When a gunshot fracture in the trochanteric region is complicated by such extensive longitudinal fissuring as to preclude excision. Experience has yet determined nothing on these points. Secondary amputations and re-amputations at the hip, in military surgery, should be performed, when, from caries, or necrosis, or chronic osteo-myelitis, following gunshot wounds, or amputations in the continuity, the patients life is in jeopardy."

The different forms of incisions which have been used are given, of which the author prefers the simple straight line. A list of bibliographical sources of information respecting this operation close the volume, on which, in the attainment and authentication of facts, the search for, and classification of statistics, and in the introduction of the illustrative plates, great labor has been expended. The practical and scientific deductions of the author are of great value to the surgical profession, while the work is, in a good sense, a complete monograph on excision of the head of the femur, for gunshot injury.

---

### *The Nation.*

It gives us pleasure to say a few commendatory words respecting this publication, which we have received from its beginning, and which furnishes us a complete history of our nation, in all its departments, contemporaneous with the occurrence of events themselves. The extraordinary ability which is requisite, in order to successfully read the history of one's own time, is recognized by every intelligent mind; and the fact that the publication has lived to enter its fifth year, is a guarantee that it will survive every future vicissitude and attain for itself lasting eminence among our nations records. Its list of contributors includes a large number of the eminent men of our nation, among whom we notice the names of

Wm. A. Hammond, Jeffries Wyman, and others distinguished in different departments of science. The favorable criticism it has received from the European press fully equals that given by our own American, and its authority is widely recognized abroad as well as at home.

---

*Politzer on the Membrana Tympani in Health and Disease.* WM. WOOD & COMPANY, New York.

This work comprises a most complete monograph upon the appearances of the membrana tympani both in health and disease. The chromo-lithographic illustrations of the membrana tympani are very valuable and very beautiful; they give to the work an attraction, and to the descriptions of disease a reality not otherwise possible. The Anatomy of this Membrane is given with great minuteness, and then the best modes of examining it, after which all the diseases to which it is liable are fully described. It is impossible to know the nature and extent of an aural affection without a knowledge of the appearances of this membrane both in health and disease. The representations here presented furnish means of determining the existence of any morbid changes which may be present, and are sufficient to enable the careful observer to arrive at very correct and satisfactory conclusions. We regard this work as very essential in the study of aural affections, since the condition of the membrana tympani, its anatomical structure and relations, with the best modes of observing it, include an important part of this field. Examination and study of the membrana tympani holds somewhat the same relation to aural diseases that examination of the retina bears to diseases of the eye. Until these structures could be examined, and their conditions positively determined, little progress was made in the study of these affections. The appearance of this book gives future promise of great progress in the diagnosis and treatment of diseases of the ear.

---

*Third Annual Report of the Metropolitan Board of Health of the State of New York.*

The district entrusted to the Metropolitan Board of Health is comprised of the Counties of New York, Kings, Westchester and Richmond, together with the towns of Newton, Flushing, and Jamaica in Queens County. The Board report that the most noticeable fact, in the mortality report of the past year, is that from one-fourth to one-half of the whole mortality is to be found among children under one year of age, and it is acknowledged that, in some portions of the city, eighty per cent. of the mortality occurs in the infant population. The rate of mortality in Brooklyn is not less than two in one thousand less than in New York. Prof. Chas. F. Chandler of the Columbia School of Mines, has been appointed Analytical Chemist to the Board during the year, and has rendered them very efficient service on several points. The abatement of the "gas nuisance" is one of the advances made during the year, and the matter is fully reported, while the processes now in use in some of the gas works to avoid the escape of noxious gasses from the purifiers is given in the chemist's report. The establishment of a Lying-in-Asylum for unfortunate women is recommended, as also the system of public drinking fouutains and baths, which has proved so beneficial in other American cities; public urinals are regarded as a necessity, and the establishment of one of these at Astor Place has been decided upon. The laying of wooden pavements to any great extent is not advised until its healthfulness and economy is ascertained. Rendering establishments have been compelled to conduct their operations in steam-tight boilers, and street disinfections, by the application of carbolic acid, was tried and approved of as being effectual. A very full and thorough account of the cattle disease that caused such excitement last year, and no less thau thirty large colored lithographic platos accompany the reports on this subject. We never have seen sanitary statistics and mortuary reports so excellently presented as they are by the lithographic plates of this volume, which is very creditable to all parties concerned in its production.

---

*Transactions of the Indiana State Medical Society.*

The last annual meeting of this Society was held at Indianapolis, in May. The address to the Society by its President, H. Field, M. D., of Indianapolis, has as its subject, "The Troubles and Responsibilities of the Medical Profession," and it gives the same a faithful representation. The subjects of the published reports are:—Why Doctors Disagree; General Anasarca; Digestive Assimilation of Medicines; and A Case of Dislocation of Femur. A discussion of Puerperal Convulsions, in which the members generally took part, is reported in full. The Society voted to present a memorial to the State Assembly, asking that a State Hospital be established at Indianapolis, separately or in conjunction with the present City Hospital.

---

We notice in the October number of the *Atlantic Monthly*, an article entitled "The Increase of Human Life," written by Dr. Edward Jarvis, of Boston. He states that the common complaint that the period of human life is gradually shortening, and that the physical condition of the race is degenerating from its pristine vigor, is unsupported by historical facts, and is furthermore contradicted by a just comparison of the records of early christian, mediæval and recent times. Only for the last fifty years have reliable records been kept very generally among nations; the oldest and most trustworthy of modern records is that which has been kept by the Canton of Geneva in Switzerland, which extends back four hundred years. Tables prepared by Ulpianus, a Roman judge, in the third century, used by the Roman courts, are the earliest account of the mathematical value of human life. From a careful review of tables of expectancy, the writer shows that they favor an increased duration of human life, that the rates of mortality are diminishing, that small-pox, measles, convulsions, fevers, teething, consumption, (sweating sickness,) etc., which formerly were dread scourges, are very much more limited in their violence and extent than they were two hundred years ago. Quite interesting statistical information respecting the sanitary condition of our own cities, as well as foreign ones, are presented in this article, which will, in its continuation, present the reasons of the better developement and sustainment of the life of men of the present stage of being.

